# Influence of antigen density and immunosuppressive factors on tumor-targeted costimulation with antibody-fusion proteins and bispecific antibody-mediated T cell response

**DOI:** 10.1007/s00262-020-02624-6

**Published:** 2020-06-05

**Authors:** Sabrina Sapski, Nadine Beha, Roland E. Kontermann, Dafne Müller

**Affiliations:** Institute of Cell Biology and Immunology, University of Stuttgart, Allmandring 31, 70569 Stuttgart, Germany

**Keywords:** Antibody-fusion proteins, Costimulation, TNFSF ligands, Cancer immunotherapy, Immunosuppression, Bispecific antibody

## Abstract

Target expression heterogeneity and the presence of an immunosuppressive microenvironment can hamper severely the efficiency of immunotherapeutic approaches. We have analyzed the potential to encounter and overcome such conditions by a combinatory two-target approach involving a bispecific antibody retargeting T cells to tumor cells and tumor-directed antibody-fusion proteins with costimulatory members of the B7 and TNF superfamily. Targeting the tumor-associated antigens EpCAM and EGFR with the bispecific antibody and costimulatory fusion proteins, respectively, we analyzed the impact of target expression and the influence of the immunosuppressive factors IDO, IL-10, TGF-β, PD-1 and CTLA-4 on the targeting-mediated stimulation of T cells. Here, suboptimal activity of the bispecific antibody at diverse EpCAM expression levels could be effectively enhanced by targeting-mediated costimulation by B7.1, 4-1BBL and OX40L in a broad range of EGFR expression levels. Furthermore, the benefit of combined costimulation by B7.1/4-1BBL and 4-1BBL/OX40L was demonstrated. In addition, the expression of immunosuppressive factors was shown in all co-culture settings, where blocking of prominent factors led to synergistic effects with combined costimulation. Thus, targeting-mediated costimulation showed general promise for a broad application covering diverse target expression levels, with the option for further selective enhancement by the identification and blockade of main immunosuppressive factors of the particular tumor environment.

**Electronic supplementary material:**

The online version of this article (10.1007/s00262-020-02624-6) contains supplementary material, which is available to authorized users.

## Introduction

Interfering with the regulatory network of the immune response has become an intensively investigated strategy for cancer immunotherapy. Besides the development of checkpoint inhibitors that antagonize coinhibitory ligands/receptors (e.g., PD-L1, PD-1, CTLA-4), there is now also increasing interest in the development of agonists for costimulatory receptors (e.g., 4-1BB, OX40, GITR, CD40) [1, 2]. Enforcing directly the immune response by costimulation might be particularly required in the presence of an immunosuppressive tumor microenvironment. Although many immunosuppressive factors have been recognized so far (e.g., TGF-β, IL-10, IDO) [3], the composition and predominance in different tumor types/stages and the role of costimulation to counteract their negative influence on the antitumor response remain largely to be investigated.

Costimulatory monoclonal antibodies are systemically active and therefore prone for the development of immune-related adverse events. A prominent example is urelumab, an agonistic 4-1BB-directed mAb that has shown dose-limiting liver toxicity in clinical studies [4]. Thus, targeting the costimulatory activity to the tumor side appears as an interesting option. This can be achieved in the form of tumor-directed antibody-fusion proteins with the ectodomain of the costimulatory ligand [5–9] or bispecific molecules targeting a tumor antigen and the respective costimulatory receptor [10]. In both cases, the costimulatory activity is targeting dependent and expected to be restricted to the tumor site. Using tumor-associated antigens as targets, discrimination between target expression in tumor and normal tissue is crucial and variation in target expression levels resulting from tumor heterogeneity has to be considered. Recently, the advantage of combining costimulation with bispecific antibodies recruiting T cells to tumor cells has been recognized and started to be explored [6, 9, 11] While bispecific antibodies targeting hematologic malignancies have successfully moved into the clinic (e.g., blinatumomab), bispecific antibodies of the same format directed against solid tumors were shown to be less effective [12]. Support by tumor-directed costimulation could improve this situation. In view of targeting competition and antigen expression heterogeneity, combination approaches targeting different antigens are emerging as interesting options. In this study, we introduce a dual-target system focusing on the tumor-associated antigens EpCAM and EGFR. Both antigens are clinical relevant targets, overexpressed, e.g., in colorectal, lung, breast, ovarian, pancreas and bladder carcinoma [13, 14]. So far, they have been successfully used as target structure for monoclonal antibodies and bispecific antibodies, although with limited therapeutic efficacy in monotherapeutic approaches [15–18]. Here, we present a combinatory approach with an EpCAM-directed bispecific antibody retargeting T cells via CD3 to tumor cells and EGFR-directed antibody-fusion proteins with costimulatory ligands of the B7 and TNF superfamily. Analysis of T cell stimulation in co-culture settings with tumor cell lines presenting different EpCAM/EGFR expression profiles demonstrated costimulatory capacity of all fusion proteins in a broad range of target expression, enhancing the effect of suboptimal bispecific antibody concentrations. In addition, the advantage of combined costimulation was demonstrated to be effective under diverse adverse tumor cell conditions and T cell stimulation was shown to be further improved synergistically by selective blockade of immunosuppressive factors.

## Materials and methods

### Materials

Antibodies were purchased from BioLegend (anti-human IL-10R(CD210), 308806; anti-human PD-1, 329912; anti-human PD-L1-APC, 329708), Miltenyi Biotech (anti-His-PE, 130-092-691), KPL (goat anti-mouse IgG H + L; 01-10-06) and R&D Systems (anti-human CD3ε, MAB100; anti-human TGF-β1,2,3, MAB1835; anti-IDO Alexa488, IC6030G; anti-His-HRP, sc-8036 HRP). Human IFN-γ (DY285), IL-2 (DY202), IL-10 (DY217B) and TGF-β (DY240) DuoSet^®^ ELISA kits were purchased from R&D Systems. A431, Colo205, HCT-116, LS174T and NCI-H460 were cultured in RPMI 1640 (Life Technologies, 11875), 10% FBS (PAN Biotech, P30-3309). Lovo and A549 were cultured in RPMI 1640, and 5% FBS and SKBR3 were cultured in DMEM (Life Technologies, 41965), 10% FBS. Human peripheral blood mononuclear cells (PBMC) were isolated from buffy coat of healthy donors (Klinikum Stuttgart, Germany) by Ficoll density gradient centrifugation (Lymphocyte Separation Medium 1077, Promocell, C-44010) and cultivated in RPMI 1640, 10% FBS. DAKO QIFIKIT (K007811-8) was purchased from Agilent Technologies.

### Generation of antibody-fusion proteins

EGFR-directed costimulatory fusion proteins were generated by replacing the antibody moiety in the previously reported scFvEDG-4-1BBL, scFvEDG-OX40L and B7.1-DbFAP [6], introducing hu225 in the scFv and Db format [19]. Sequences are indicated in Supplementary Table 2. To generate the bispecific antibody, EpCAM-directed humanized MOC-B VH and VL [20] were used to create scDbEpCAMxCD3, based on the model of scDbFAPxCD3 [6]. Recombinant protein was produced by transient transfection in HEK-293-6E cells (NRC Biotechnology Research Institute, Canada) according to the standard protocol of the cell line provider, followed by purification via immobilized metal ion affinity chromatography (IMAC) as described previously [6].

### Size-exclusion chromatography

Purified protein was analyzed by size-exclusion chromatography using a BioSep-SEC-S2000 or TSK-GEL G3000SWXL column and 0.1 M Na_2_HPO_4_/NaH_2_PO_4_, 0.1 M Na_2_SO_4_, pH 6.7 as mobile phase at a flow rate of 0.5 ml/min. Standard proteins were thyroglobulin (669 kDa), β-amylase (200 kDa), bovine serum albumin (67 kDa) and carbonic anhydrase (29 kDa).

### Binding analysis

For the flow cytometry analysis, 2 × 10^5^ target cells/well were incubated with the respective fusion protein for 1 h at 4 °C. Bound protein was detected by PE-conjugated anti-hexahistidyl-tag antibody, respectively. Fluorescence was measured by MACSQuant Analyzer10/VYB (Miltenyi Biotech), and data were analyzed using FlowJo (Tree Star). Relative mean fluorescence intensity (MFI) = (MFI_sample_ − (MFI_detection system_ − MFI_cells_))/MFI_cells_.

For the ELISA analysis, EGFR-Fc (100 ng/well in PBS) was coated on 96-well ELISA plates overnight at 4 °C. After blocking with 2% (w/v) non-fat dry milk/PBS, plates were incubated for 1 h at RT with the corresponding antibody-fusion proteins. Bound fusion protein was detected via anti-hexahistidyl-tag-HRP antibody, using 3,3′,5,5′-tetramethylbenzidine (TMB) substrate. Absorption (450 nm) was measured in a Tecan infinite M200 reader.

### Costimulatory assays

2 × 10^4^ target cells/well were seeded in 96-well plates. In parallel, PBMCs were thawed and cultured overnight. The next day, target cells were preincubated for 1 h at RT with the recombinant fusion proteins before the addition of blocking monoclonal antibody, cytokines or inhibitors, according to the corresponding assay design. After 1 h incubation, 2 × 10^5^ PBMCs/well were added. Supernatants were harvested after 24 or 48 h and concentration of IL-2 or IFN-γ, respectively, determined by sandwich ELISA (DuoSet ELISA kit) according to the manufacturer’s instruction.

### Analysis of EpCAM/EGFR cell surface expression

The expression level of EpCAM and EGFR on the cell surface was determined by flow cytometry using the QIFIKIT (Agilent Technologies). 2 × 10^5^ target cells/well were incubated (1 h, 4 °C) with mouse anti-EGFR or EpCAM antibody, respectively, at saturating concentrations. After washing with PBA, cells were incubated (1 h, 4 °C) with FITC-labeled anti-mouse antibody at saturation. After washing, fluorescence was measured by flow cytometry and antigen density determined from a standard curve obtained by beads coated with defined amounts of mouse IgG.

### Measurement of immunosuppressive factors

2 × 10^4^ tumor target cells/well were seeded in 96-well plates and incubated the next day with or without 2 × 10^5^ stimulated PBMCs/well (0.1 µg/ml anti-CD3 mAb cross-linked by goat anti-mouse IgG antibodies at a molar ratio of 1:3) for 48 h. Supernatant and PBMCs were removed, and tumor cells were analyzed for PD-L1 and IDO expression by flow cytometry. PD-L1 expression was detected by extracellular staining with anti-PD-L1-APC antibody. IDO was detected by intracellular staining. Therefore, cells were treated with 4% PFA and FOXP3 Perm buffer (Biolegend, 421402) and stained with anti-IDO AlexaFluor488 mAb. Concentration of IL-10 and TGF-β was determined in cell-free supernatant by sandwich ELISA according to the manufacturer’s instruction.

### Statistical analysis

Unless otherwise stated, all data are represented as mean ± S.D. of three independent experiments. Block shift correction was performed according to the formula: *X*ʹ_*n*_ = *X*_*n*_ − (*Y*_n_ − *Y*) with *X*ʹ_*n*_ being the corrected value of *X* from the experiment *n*, *Y* the average of the *X* values from all experiments performed and *Y*_*n*_ the average of the duplicate values of *X* from experiment *n*. Statistical significance was determined using one-way ANOVA followed by Tukey’s posttest (Graphpad Prism, Graphpad Software Inc., La Jolla, USA). *P* values below 0.05 were considered statistically significant (***P < 0.001, **P < 0.01, *P < 0.05).

## Results

**Fig. 1:**
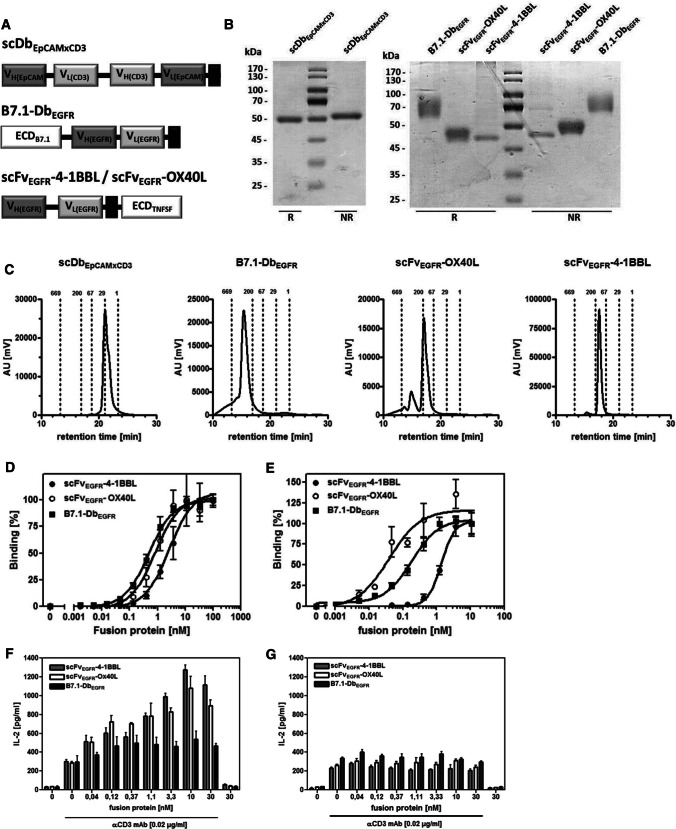
Bispecific antibody and costimulatory antibody-fusion proteins. **a** Modular schema of the recombinant proteins applied in the combinatorial settings. ECD, extracellular domain; *V*_H/L_, variable region of the heavy/light antibody chain; black box, hexahistidyl-tag **b** 12% SDS-PAGE analysis of fusion proteins (3 µg/lane) under reducing (R) and non-reducing (NR) conditions. Coomassie staining. **c** Size-exclusion chromatography analysis of fusion proteins on BioSep-SECS2000/ TSK-GEL G3000SWXL columns. Mobile phase PBS. **d** Binding analysis to EGFR-Fc in ELISA. Bound fusion protein was detected via anti-hexahistidyl-tag-HRP antibody. **e** Binding analysis to EGFR on A431 cells by flow cytometry. Bound fusion protein was detected by anti-hexahistidyl-tag-PE antibody. **f**, **g** Activity of costimulatory fusion proteins in target-bound and soluble form. Fusion proteins were incubated in the presence (**f**) or absence (**g**) of A431 (EGFR +) target cells with cross-linked anti-CD3 mAb and PBMCs for 24 h. IL-2 release was determined in the supernatant by sandwich ELISA. Graphics show mean ± SD, *n* = 3 (duplicates in each assay), and block-shift correction

The experimental setting for the combinatorial approach comprises on the one hand a bispecific antibody directed against EpCAM and CD3, thus retargeting T cells to tumor cells, inducing initial T cell stimulation in a tumor cell-directed, but MHC-independent manner. On the other hand, costimulatory antibody-fusion proteins composed of an EGFR-specific antibody part and the extracellular domain of costimulatory ligands of the B7 superfamily (B7.1) and TNF superfamily (4-1BBL, OX40L) are added. Antibody-mediated targeting leads here to the cell surface presentation of the costimulatory ligand, mimicking its physiological active transmembrane form, enhancing and modulating the T cell stimulation initiated by the bispecific antibody. Targeting different tumor-associated antigens (EpCAM/EGFR) on the tumor cell is expected to support the combinatorial approach by avoiding competition between the fusion proteins mediating the first and the costimulatory signal, respectively. The bispecific antibody was generated in the single-chain diabody format (scDbEpCAMxCD3), thus being monovalent for each specificity (Fig. 1a). Antibody-fusion proteins composed of the antibody scFv and 4-1BBL or OX40L present as homotrimeric molecules, due to trimerization via the TNFSF ligand, while the antibody-fusion protein composed of the antibody Db and the B7.1 ligand presents as homodimeric molecule, due to the dimerization inherent of the diabody format (Fig. 1a). All recombinant proteins were produced in HEK293-6E cells and purified via hexahistidyl-tag by IMAC. SDS-PAGE analysis showed single bands correlating to the calculated molecular mass of the single chains of scDbEpCAMxCD3 (54 kDa), scFvEGFR-4-1BBL (47 kDa), scFvEGFR-OX40L (43 kDa) and B7.1DbEGFR (52 kDa), respectively, taking into consideration that OX40L and B7.1 are strongly glycosylated (Fig. 1b). Size-exclusion chromatography showed a main peak for all costimulatory fusion proteins, where a smaller apparent molecular mass is typical for the single-chain diabody format (personal observation) and a higher apparent molecular mass of B7.1-DbEGFR and scFvEGFR-OX40L is attributable to glycosylation. A secondary peak in the case of scFvEGFR-OX40L indicated the presence of a small hexamer fraction (Fig. 1c). Functional analysis of the costimulatory antibody-fusion proteins showed binding to recombinant EGFR in ELISA (Fig. 1d) and EGFR expressed on cells by flow cytometry (Fig. 1e). In ELISA, binding capacity of scFvEGFR-4-1BBL (EC_50_ = 2.62 ± 0.90 nM) was three- and fivefold reduced in comparison with scFvEGFR-OX40L (EC_50_ = 0.84 ± 0.20 nM) and B7.1-DbEGFR (EC_50_ = 0.49 ± 0.10 nM), while cell binding capacity of scFvEGFR-4-1BBL (EC_50_ = 1.41 ± 0.16 nM) was approximately 7- to 28-fold reduced in comparison with B7.1-DbEGFR (EC_50_ = 0.18 ± 0.01) and scFvEGFR-OX40L (EC_50_ = 0.05 ± 0.04 nM), respectively. However, in co-culture assays with A431 cells and PBMCs, in the presence of a suboptimal concentration of cross-linked anti-CD3 mAb, the costimulatory activity of target-bound fusion proteins was similar for scFvEGFR-4-1BBL and scFvEGFR-OX40L and less pronounced for B7.1-DbEGFR (Fig. 1f). In addition, the costimulatory nature of the fusion protein activity was confirmed by their incapacity to induce T cells activation by their own. Also, targeting dependency of the activity was confirmed for all costimulatory antibody-fusion proteins, since none of them showed activity in soluble form, i.e., in the absence of target cells (Fig. 1g). Thus, for all EGFR-directed antibody-fusion proteins it was corroborated that target binding was required and binding capacity sufficient to display ligand activity.

**Fig. 2:**
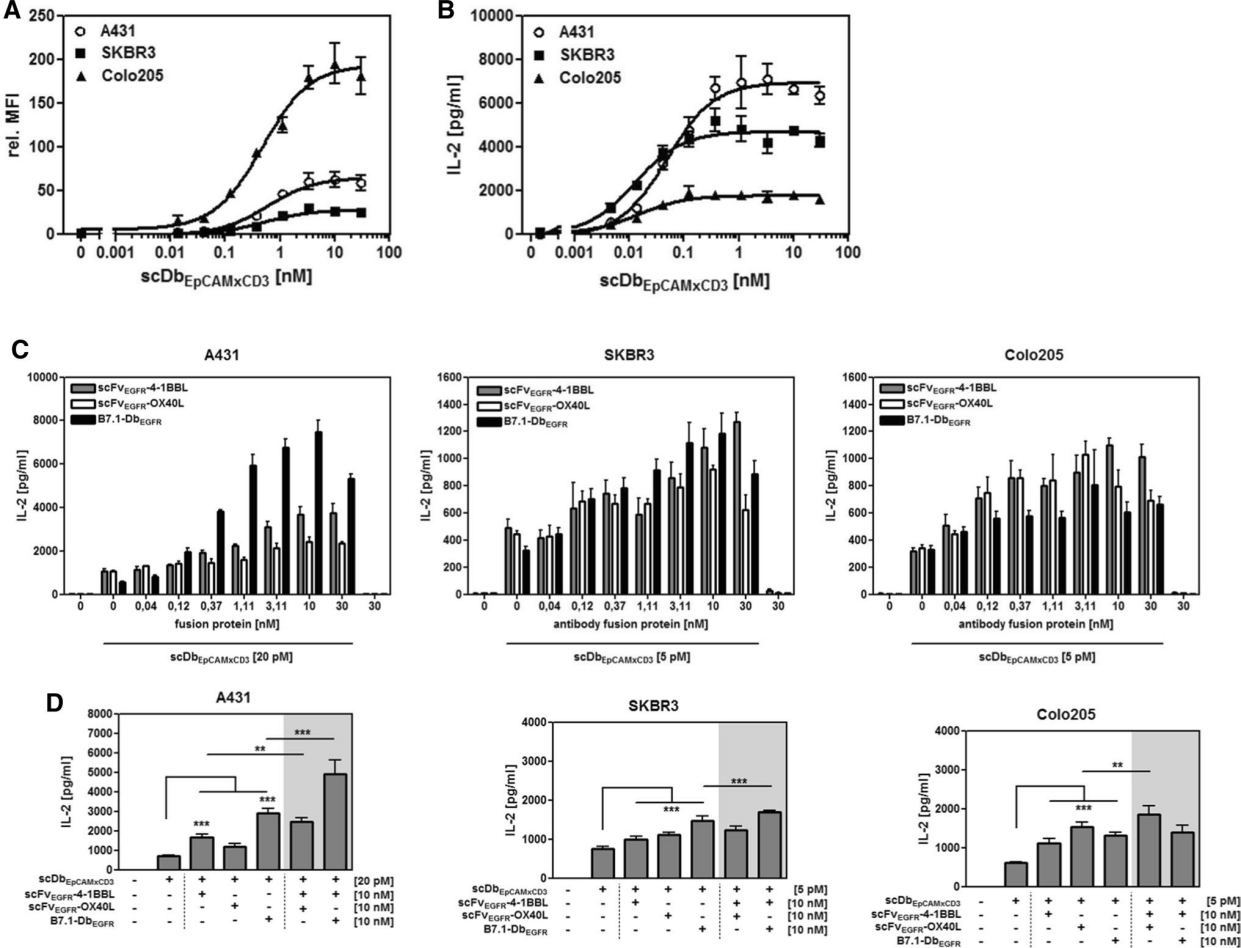
Costimulatory activity of the antibody-fusion proteins targeting cells with different EGFR expression levels. **a** Binding of scDbEpCAMxCD3 to EpCAM on A431, SKBR3 and Colo205 cells determined by flow cytometry. Bound bispecific antibody was detected by anti-hexahistidyl-tag-PE antibody. **b** scDbEpCAMxCD3-mediated T cell activation. Target cells (A431, SKBR3 and Colo205 cells) were incubated with the bispecific antibody and PBMCs for 24 h. IL-2 release was determined in the supernatant by sandwich ELISA. **c**, **d** Costimulatory activity of antibody-fusion proteins on target cells with high (A431), medium (SKBR3) and low (Colo205) EGFR expression level. Costimulatory fusion proteins were either individually titrated (**c**) or combined at a concentration of 10 nM (**d**) and incubated on target cells in the presence of the bispecific antibody (suboptimal concentration) and PBMCs. After 24 h, IL-2 concentration in the supernatant was measured by sandwich ELISA. Graphics show mean ± SD, *n* = 3 (duplicates in each assay), block-shift correction, and **p < 0.01; ***p < 0.001

Next, we assessed the costimulatory activity of the antibody-fusion proteins in combination with the bispecific antibody. Here, the influence of the antigen expression was addressed using settings with high (> 572,000 molecules/cell) to rather low (~ 5,000 molecules/cell) EGFR levels co-expressed with high (> 150,000 molecules/cell) EpCAM levels. Therefore, the tumor cell lines A431, SKBR3 and Colo205 were chosen (Supplementary Table 1). First, binding of scDbEpCAMxCD3 to these tumor cell lines was determined by flow cytometry (Fig. 2a) and T cell activation measured in co-culture assays by IL-2 release (Fig. 2b). The bispecific antibody demonstrated to be effective in EpCAM binding and T cell activation in a concentration-dependent manner on all three cell lines. In the following assays, suboptimal concentrations (EC_25_) of bispecific antibody as determined in the T cell activation assay were applied in combination with the costimulatory fusion proteins. In all three co-culture settings, the costimulatory fusion proteins showed to enhance the bispecific antibody-induced T cell stimulation in a concentration-dependent manner (Fig. 2c). Similar costimulatory capacity was shown for scFvEGFR-4-1BBL (approx. threefold) and scFvEGFR-OX40L (approx. 2.5-fold) in all three co-culture settings, regardless of the EGFR expression levels. In contrast, the costimulatory effect observed for B7.1-DbEGFR decreased with reduced EGFR expression levels, resulting in approx. 13-, 3.7- and 2.5-fold stimulation enhancement in the settings with A431 (> 572.000 EGFR/cell), SKBR3 (~ 33,000 EGFR/cell) and Colo205 (~ 5,000 EGFR/cell), respectively. In addition, the potential to further increase the stimulation by combination of scFvEGFR-4-1BBL with scFvEGFR-OX40L or B7.1-DbEGFR at saturating concentration (10 nM) was analyzed (Fig. 2d). In the presence of high EGFR expressing A431 cells, the combination of scFvEGFR-4-1BBL and B7.1-DbEGFR was particularly effective, while in the presence of low EGFR expressing Colo205 cells the combination of scFvEGFR-4-1BBL and scFvEGFR-OX40L showed to be advantageous. Thus, targeting tumor cells expressing EGFR in a range of approximately 5,000–600,000 EGFR/cell, all three antibody-fusion proteins showed to be costimulatory active. Only the efficacy of B7.1-DbEGFR was clearly related to the EGFR expression, while scFvEGFR-4-1BBL and scFvEGFR-OX40L performed similarly consistent in this range. Accordingly, combined application of scFvEGFR-4-1BBL with B7.1-DbEGFR distinguished at high EGFR expression levels, while the combination of scFvEGFR-4-1BBL with scFvEGFR-OX40L prevailed effective at a low EGFR expression level.

**Fig. 3:**
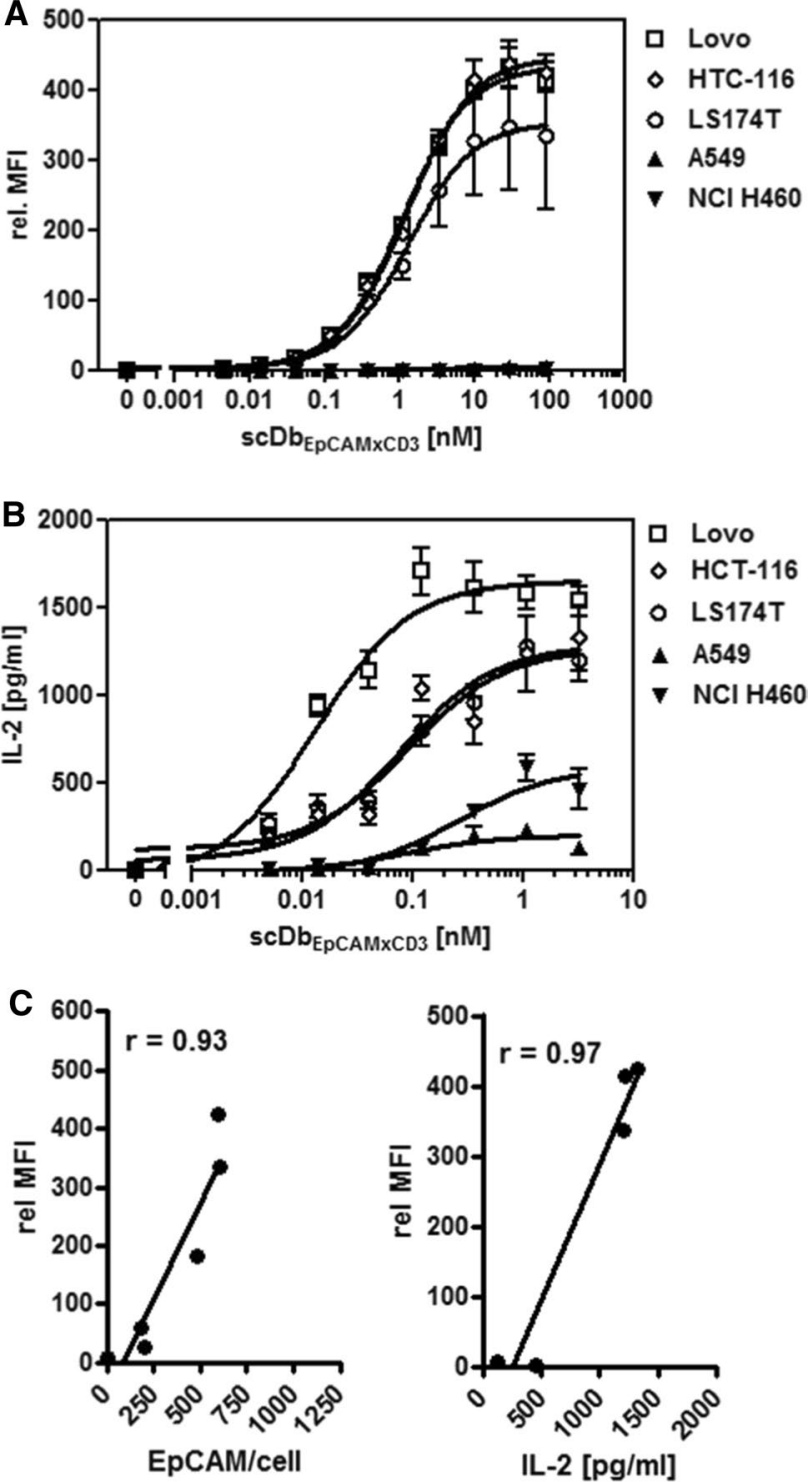
Binding and retargeting activity of scDbEpCAMxCD3 on colon and lung cancer cell lines with different EpCAM expression levels. **a** Binding of scDbEpCAMxCD3 to target cells analyzed by flow cytometry. Bound scDbEpCAMxCD3 was detected by anti-hexahistidyl-tag-PE antibody. **b** scDbEpCAMxCD3-mediated T cell activation. Target cells were incubated with the bispecific antibody and PBMCs for 24 h. IL-2 release into the supernatant was measured by sandwich ELISA. **c** Correlation of maximal bispecific antibody binding to target cells (highest relative MFI) and the EpCAM expression level on target cells (EpCAM/cell) or the maximal T cell activation capacity (maximal IL-2 release) in corresponding co-culture settings. Graphics show mean ± SD, *n* = 3 (duplicates in each assay), and block-shift correction

**Fig. 4:**
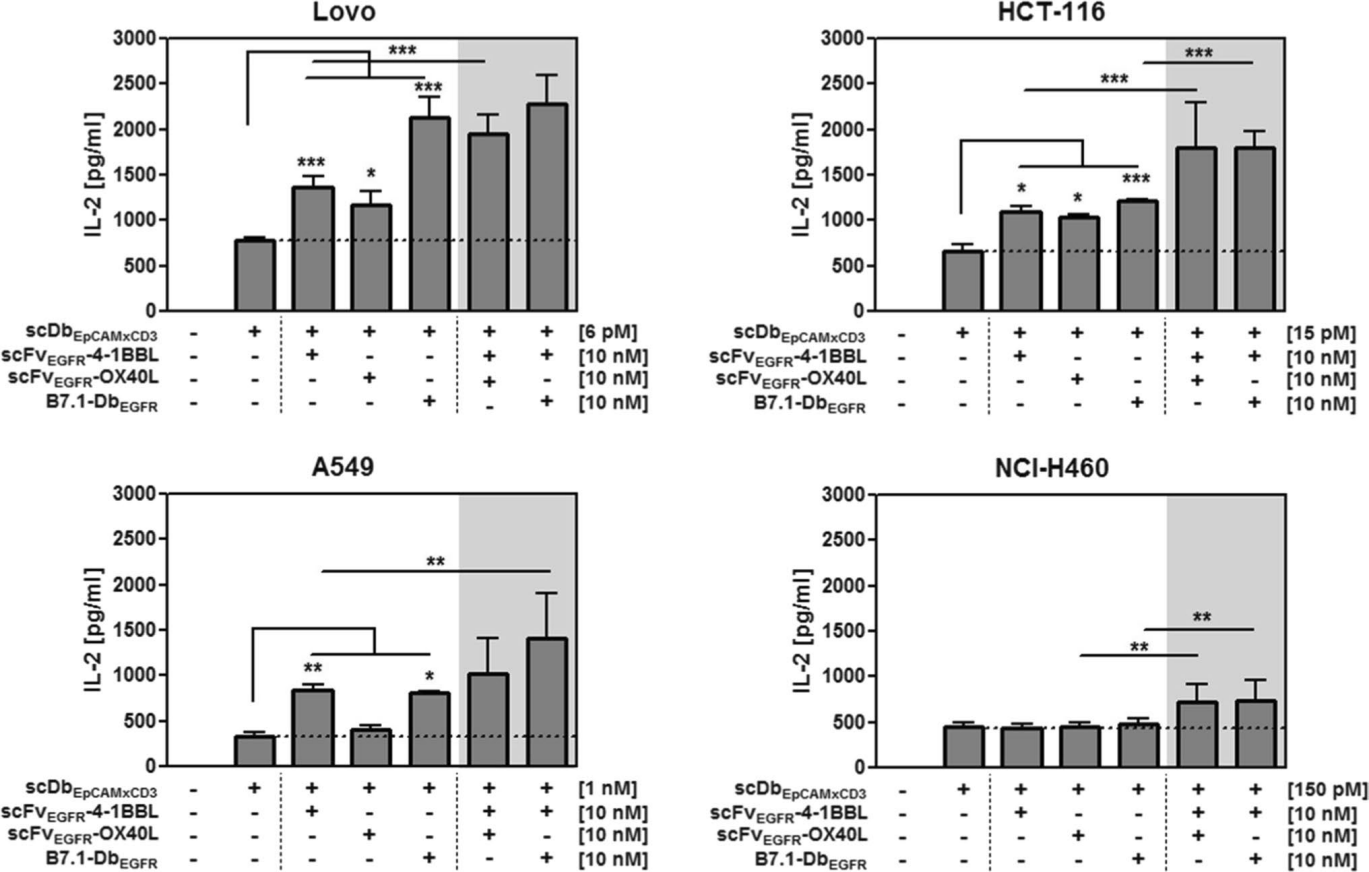
Costimulatory effect of fusion protein combinations on bispecific antibody-mediated T cell activation in the context of different EpCAM/EGFR expression levels. Target cells differing in expression levels of EpCAM and EGFR were incubated with costimulatory fusion proteins either alone or in combination with the presence of bispecific antibody (suboptimal concentration) and PBMCs. T cell activation was determined by measuring IL-2 release into the supernatant by sandwich ELISA. Graphics show mean ± SD, *n* = 3 (duplicates in each assay), block-shift correction, and *P < 0.05; **P < 0.01; ***P < 0.001

Next, the analysis was extended and focused on the colon carcinoma cell lines Lovo (> 769,000 EpCAM/cell, ~ 182,000 EGFR/cell), LS174T (~ 602,000 EpCAM/cell, ~ 29,000 EGFR/cell) and HTC-116 (~ 591,000 EpCAM/cell, ~ 17,000 EGFR/cell) characterized by particular high EpCAM and high to moderate EGFR expression levels and the lung carcinoma cell lines A549 (~ 8,000 EpCAM/cell, ~ 80,000 EGFR/cell) and NCI-H460 (< 2,000 EpCAM/cell, ~ 42,000 EGFR/cell) presenting rather low EpCAM and moderate EGFR expression levels (Supplementary Table 1). Also here, scDbEpCAMxCD3-mediated binding to the tumor cells (Fig. 3a) and activation of T cells (Fig. 3b) were initially assessed as previously described. A positive correlation between EpCAM expression levels and maximal antibody binding (*r* = 0.93) as well as maximal antibody binding and maximal T cell activation (*r* = 0.97) was observed (Fig. 3c). Next, T cell stimulation was assessed in experimental settings of co-cultures of the respective cell lines with PBMCs in the presence of the bispecific antibody (EC_25_/EC_100_) and the individual or combined costimulatory antibody-fusion proteins (10 nM) in terms of IL-2 release (Fig. 4). In the colon carcinoma cell lines with high EpCAM expression levels, the initial stimulation provided by the bispecific antibody was efficiently enhanced by all three costimulatory fusion proteins targeted to highly and moderately expressed EGFR. Also here, scFvEGFR-4-1BBL and scFvEGFR-OX40L demonstrated similarly consistent activity that was further increased by their combined application. B7.1-DbEGFR showed to be particularly effective as single costimulatory agent at high EGFR density (Lovo ~ 182,000 EGFR/cell) and to be an effective combination partner to scFvEGFR-4-1BBL at moderate EGFR density (HCT-116 ~ 17,000 EGFR/cell). In the lung cancer cell lines, the moderate EGFR expression exceeded clearly the EpCAM expression. Costimulatory activity was also shown under these conditions, where at a very low EpCAM level (NCI-H460 < 2,000 EpCAM/cell) the effect of the bispecific antibody could not be enhanced anymore by individual costimulation, but was still increased by the combined application of scFvEGFR-4-1BBL with B7.1-DbEGFR or scFvEGFR-OX40L, respectively. Thus, effective costimulation was achieved at different EpCAM/EGFR expression ratios and in this context the benefit of combined costimulation was demonstrated. The potential to increase scDbEpCAMxCD3-mediated tumor cell killing was shown for scFvEGFR-4-1BBL and B7.1-DbEGFR in a setting with Lovo cells. However, further significant enhancement by their combination was not observed (Supplementary Fig. 1).

**Fig. 5:**
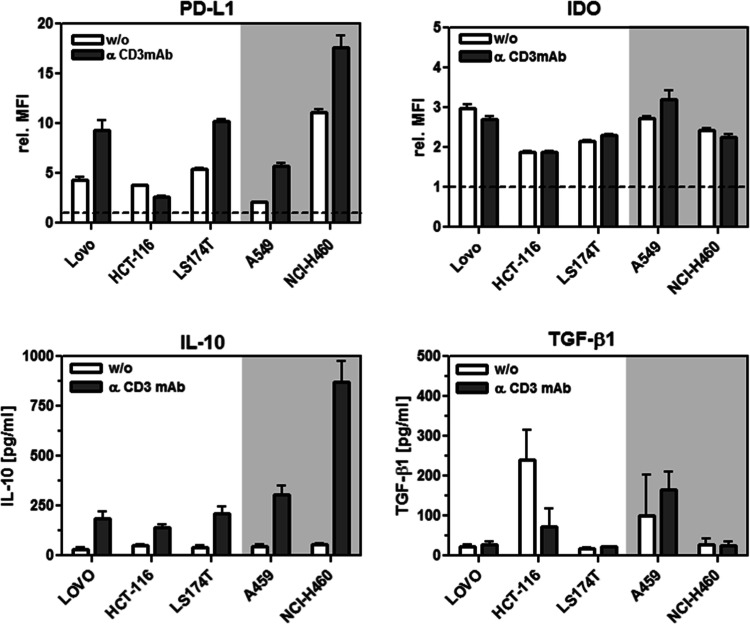
Expression of immunosuppressive factors in co-cultures of EpCAM + EGFR + tumor cells (Lovo, HCT-116, LS174T, A549, NCI-H460) and unstimulated or stimulated (0.1 µg/ml cross-linked anti-CD3 mAb) PBMCs. Tumor cell expression of PD-L1 and IDO was determined by flow cytometry. The presence of IL-10 and TGF-β in the supernatant was quantified by sandwich ELISA. Graphics show mean ± SD, *n* = 3 (duplicates in each assay), and block-shift correction

**Fig. 6:**
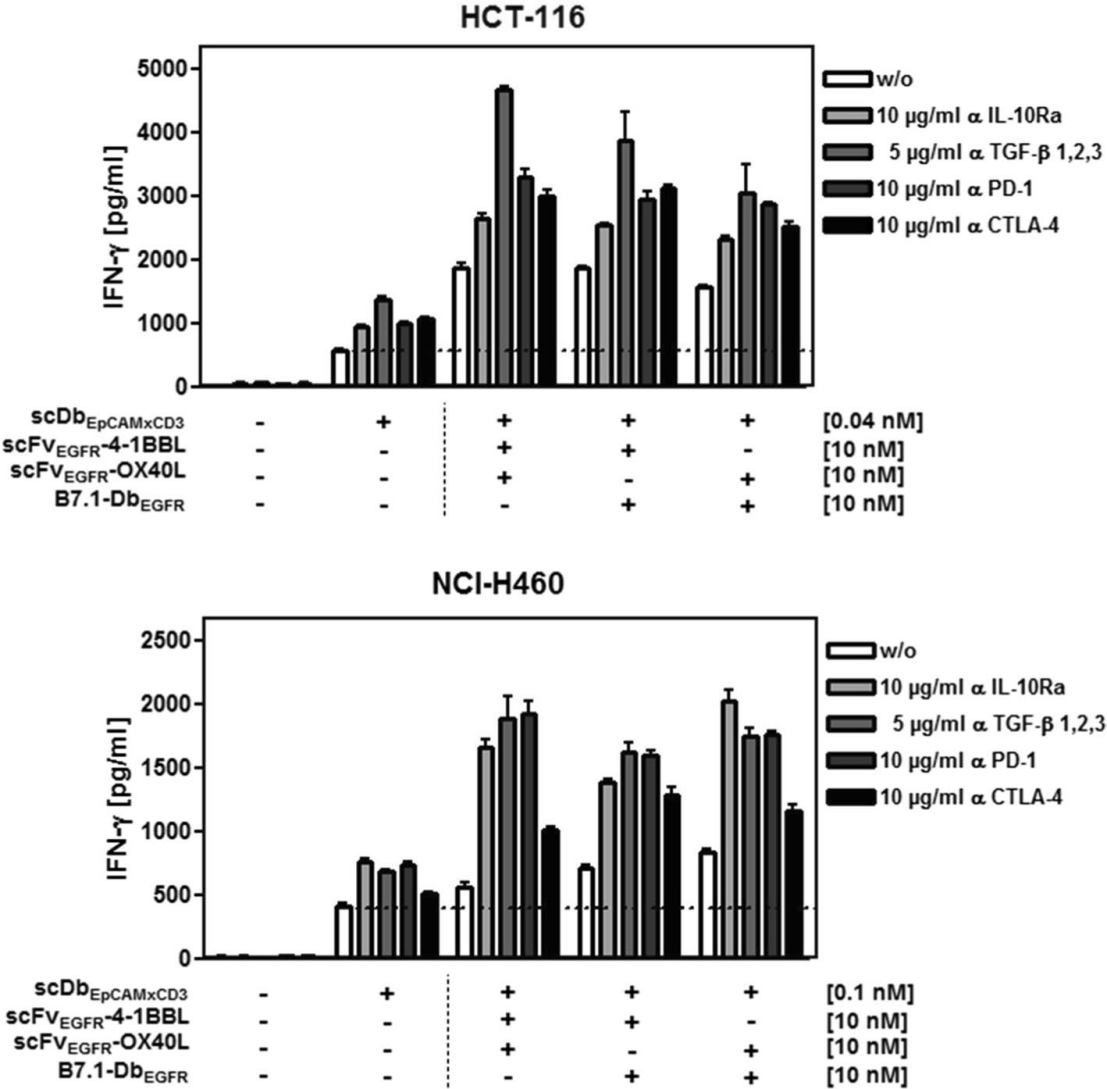
Blocking immunosuppressive factors synergizes with combined costimulation of the antibody-fusion proteins, enhancing the bispecific antibody-mediated T cell stimulation. Target cells (HCT-116 and NCI-H460) were incubated with bispecific antibody (suboptimal concentration) and combinations of costimulatory antibody-fusion proteins for 1 h. Then, antagonistic IL-10Ra mAb, anti-TGF-β1,2,3 mAb, anti-PD-1 mAb and anti-CTLA-4 mAb were applied for another hour to block the activity of the respective cytokines and inhibitory receptors before the addition of PBMCs. After co-culture with PBMCs for 48 h, supernatants were removed and concentrations of IFN-γ measured by sandwich ELISA. Graphics show mean ± SD, *n* = 3 (duplicates in each assay), and block-shift correction

Since T cell stimulation can also be expected to be influenced by the presence of immunosuppressive factors, the expression of PD-L1, IDO, IL-10 and TGF-β was determined for all tumor cell lines in the presence and absence of stimulated PBMCs (Fig. 5). Indeed, expression of PD-L1 and IDO was detected to different degrees on and in all five cell lines, respectively, whereat the presence of activated PBMCs clearly enhanced the levels of PD-L1 on most tumor cell lines in contrast to the IDO expression that remained similar. IL-10 was consistently present in all co-cultures with activated PBMCs, standing out the high levels observed in the co-culture with NCI-H460. TGF-β expression showed comparatively higher levels in the cultures of HCT-116 and A459 cells either in the presence or absence of activated PBMCs. Next, in the context of a combinatory approach, we analyzed the impact of IL-10, TGF-β, PD-1 and CTLA-4 on T cell stimulation (IFN-γ release) by blocking these factors in our experimental setting with corresponding antagonistic antibodies (Fig. 6). In the co-culture setting with HCT-116/PBMCs, the activation induced by the bispecific antibody only was enhanced by blocking the individual immunosuppressive factors. However, these effects were in general smaller than the enhancement obtained by the application of the costimulatory antibody-fusion proteins pairs, indicating their potential to drive the T cell response under these unfavorable conditions. Furthermore, the costimulatory effect could be significantly increased by the additional blockade of TGF-β, PD-1 or CTLA-4. The combinatorial effects achieved by checkpoint inhibition were in general similar for PD-1 and CTLA-4. In combination with scFvEGFR-4-1BBL and scFvEGFR-OX40L, blocking TGF-β was in particular effective, coinciding with a high TGF-β expression profile observed. In the co-culture setting with NCI-H460/PBMCs, the costimulatory effect achieved by the fusion protein pairs was in the same range than the effect obtained by blocking the individual inhibitory factors. However, combination of both, dual costimulation and suppression of inhibitory factors, led in general to a significant enhancement of T cell stimulation. Additional blocking of IL-10, TGF-β or PD-1 was similar effective, while CTLA-4 inhibition was clearly less effective than PD-1 inhibition, coinciding with a high PD-L1 expression observed. Overall stimulation achieved in the NCI-H460/PBMCs co-culture was lower than in the HCT-116/PBMCs co-culture, coinciding with a lower EpCAM target expression level for the first signal, indicating a crucial role in limiting the extension of the response. In summary, combined costimulation was demonstrated to support T cell stimulation in the presence of mixed constellations of the common immunosuppressive factors PD-L1, IDO, IL-10 and TGF-β. Furthermore, the costimulatory effect could be significantly increased under these conditions by selective inhibition of suppressive factors according to their expression profile, suggesting further opportunities for future combination therapies.

## Discussion

Current developments of bispecific antibodies and CAR-T cells targeting Her2 and EGFR, respectively, have shown that tumor selectivity can be enhanced by lowering the affinity and enforcing the avidity of the antibody; thus, T cell triggering becomes only effective at high antigen density [21, 22]. However, a heterogeneous target expression or a suboptimal prevalence of target expression in the tumor might still limit this approach. Indeed, CEA expression heterogeneity and plasticity were reported to confer resistance to cibisatamab, an optimized CEAxCD3 bispecific antibody, in patient-derived colorectal cancer organoids [23]. Interestingly, recent studies with CD38-directed CAR-T cells showed that low scFv affinity could be compensated by costimulation via CD28 and 4-1BB to rescue and enforce the antitumor effect, pointing out the potential of costimulation in a combinatorial design [24]. Here, we addressed the issue by proposing a combinatorial approach targeting two different tumor-associated antigens (EpCAM and EGFR) with a bivalent bispecific antibody and multivalent costimulatory antibody-fusion proteins with affinities in the lower nanomolar range, respectively. Co-expression of the targets at the tumor site is expected to allow the application of suboptimal bispecific antibody dosage that combined with local costimulation lead to an effective T cell activation confined to the tumor site. In co-culture settings with high EpCAM/varying EGFR expressing target cells and PBMCs, we observed that T cell stimulation induced by suboptimal concentration of the bispecific antibody could be enhanced by all costimulatory antibody-fusion proteins in a broad range of EGFR expression. Target expression level influenced in particular the costimulatory efficiency via CD28, but not via 4-1BB or OX40. This is consistent with CD28 costimulation playing a pivotal role in primary T cell activation, orchestrating membrane raft trapping at the immunological synapse and direct quantitative TCR signal amplification [25]. On the other hand, costimulation by 4-1BB and OX40, upregulated after T cell activation, plays a main role in subsequent functional persistence and survival of T cells and is involved in the memory T cell response [26]. Independency of CD28 costimulation and even in some circumstances of TCR signaling has been reported [27–30]. Mechanistically, it has been hypothesized that after ligand encounter 4-1BB and OX40 receptors form high-order clusters in lipid rafts, allowing the accumulation of PI3K and Akt in concentrated depots in close proximity to the TCR signalosome, thus enhancing and sustaining then PI3K–Akt pathway activation [31, 32]. Hence, convergence with TCR signaling occurs at a later time point and mechanistically different from CD28. Target density appears here less critical to assure consistent costimulatory support, whereupon targeting is further facilitated by the multivalency of the scFv-4-1BBL/Ox40L fusion protein format. Accordingly, in terms of synergy, at high EGFR target expression, the combination of B7 and 4-1BBL was in particular effective, while at low EGFR target expression the combination of 4-1BBL and OX40L resulted advantageous. Those combinations have been described previously as promising in boosting T cell expansion, effector function and antitumor immunity, e.g., in the design of artificial antigen-presenting cells [33], combined agonistic mAb approaches [34] and antibody-fusion proteins [6, 7], respectively. Here, in the two-target approach, the potential of combined costimulation was in particular advantageous enforcing T cell stimulation at favorable high dual-target expression, but enabled also local stimulation, although to lesser degree, under more heterogeneous co-expression conditions. Thus, combined targeted costimulation might contribute to extend the tumor-selective range of action. Likewise, in another study, enhanced “tumor sensing” has been shown by a two-target approach with T cells transduced with a chimeric antigen receptor (CAR) of suboptimal activation properties binding to PSCA and a chimeric costimulatory receptor (CD28/4-1BB) binding to PSMA, where co-transduced T cells destroyed only tumor cells co-expressing both antigens [35].

Separate delivery of two tumor-targeted reagents becomes challenging in vivo and will certainly require adjustments coordinating the individual pharmacokinetic and pharmacodynamic properties. However, recent preclinical animal studies combining bispecific antibodies (TAAxCD3) with tumor-directed costimulation by bispecific antibodies or antibody-fusion proteins have demonstrated that strategies targeting different epitopes on the same tumor target, two different targets on the same tumor cell or different targets on tumor cell and tumor stroma cell, respectively, are feasible in vivo, improving the therapeutic effect [9, 36].

Even though a positive correlation between the target expression for the bispecific antibody and the extent of T cell stimulation was observed, we also showed that the outcome was additionally modulated by multiple factors accounting for a T cell hostile environment (e.g., IDO, TGF-β, IL-10, PD-1, CTLA-4). Accordingly, enhanced efficacy of diverse bispecific antibodies (TAAxCD3) in combination with checkpoint inhibitors has also been reported by other groups and corresponding clinical trials are in progress [37]. In our co-culture settings, almost all factors investigated were present to some degree after T cell activation, reflecting the complexity of the environment encountered by the immunomodulatory network [3]. Here, we could show that the contribution of individual factor inhibition was only minor in comparison with the strong and often synergistic effect achieved by their combination with costimulation. This emphasizes the potential of costimulation in the design of combinatory approaches, which is also reflected by the increasing number of clinical trials involving a costimulatory compound [2]. Blocking TGF-β in the presence of 4-1BB costimulation resulted particularly effective in the co-culture setting with HCT-116 cells that showed comparatively high expression of TGF-β. Although costimulation by 4-1BB has demonstrated the capacity to oppose TGF-β activity, e.g., by enforcing T cell stimulation enhancing IFN-γ release [38], abrogating the suppression of cytotoxic T cell differentiation [39] and inhibiting the conversion of conventional CD4 + T cells into iTregs [40], TGF-β has also been reported to reduce the expression of 4-1BB [41] and to contribute to the resistance to anti-4-1BB therapy by inducing CD73 expression on human CD8 + T cells [42]. Therefore, additional TGF-β blockade to 4-1BB costimulation seems to be indicated for optimal results. Also, the combination with OX40 costimulation holds potential, supported by the observation that treatment with an agonistic OX40 antibody and a TGF-β receptor signaling inhibitor (SM16) led to tumor regression in diverse mouse models [43, 44]. In the co-culture setting with the high PD-L1 expressing NCI-H460 cell line, combination of costimulatory fusion proteins with PD-1 checkpoint inhibition resulted clearly more effective than CTLA-4 checkpoint inhibition. This coincides with the postulated role of PD-L1 as potential biomarker for the prediction of PD-1/PD-L1 checkpoint inhibitor efficacy [45]. In several preclinical studies, enhanced treatment effects have been reported for the combination of PD-1/PD-L1 axis inhibition with costimulation by 4-1BB and OX40, respectively [46, 47]. Now, these combinations have also started to being explored in clinical studies (NCT02554812). Furthermore, recognizing the important role of TGF-β and PD-L1 in tumor resistance, a bifunctional fusion protein blocking simultaneously the TGF-β and PD-1 pathway has been developed (M7824), enabling immune cell infiltration and tumor regression in preclinical studies, moving into first clinical trials [48]. In another study combination of TGF-β blockade, PD-1 inhibition and 4-1BB costimulation were demonstrated to be particularly effective in enhancing abscopal effects in mice [49]. Thus, for the design of immunotherapeutic strategies, targeted costimulation holds great potential as combination partner not only for bispecific antibodies but also for the increasing variety of anti-immunosuppressive agents. For precision medicine, options of selectively driving the antitumor immune response might become pivotal to reach the right balance for cancer therapy.

## Electronic supplementary material

Below is the link to the electronic supplementary material.

Supplementary file1 (PDF 82 kb)

Supplementary file2 (PDF 72 kb)

Supplementary file3 (PDF 111 kb)

## Abbreviations

Db: Diabody
EGFR: Epidermal growth factor receptor
EpCAM: Epithelial cell adhesion molecule
IDO: Indoleamine-2,3-dioxygenase
scDb: Single-chain diabody
scFv: Single-chain fragment variable
TNFSF: Tumor necrosis factor superfamily
