# Supplementary material for: Influence of antigen density and immunosuppressive factors on tumor-targeted costimulation with antibody-fusion proteins and bispecific antibody-mediated T cell response
Source: Cancer Immunol Immunother. 2020 Jun 5;69(11):2291–303. doi: 10.1007/s00262-020-02624-6 (PMC7568714; doi:10.1007/s00262-020-02624-6)
Supplement: Supplementary file 1 — Supplementary file1 (PDF 82 kb) [file 262_2020_2624_MOESM1_ESM.pdf]

Supplementary Table 1

Expression levels of EpCAM and EGFR on tumor cell lines determined by QIFIKIT®

cell line	EpCAM (molecules/cell)	EGFR (molecules/cell)
**A431**	180.263 ± 62.217	> 572.000
**SKBR3**	198.409 ± 19.113	32.922 ± 7.197
**Colo205**	486.350 ± 10.962	5.293 ± 2.616
**Lovo**	>769.000	181.649 ± 72.538
**LS174T**	602.122 ± 161.152	28.623 ± 3.528
**HCT-116**	591.491 ± 57.758	17.251 ± 1.373
**A549**	7.965 ± 2.143	80.061 ± 1.446
**NCI-H460**	< 2.000	41.887 ± 10.286
