# Supplementary material for: Influence of antigen density and immunosuppressive factors on tumor-targeted costimulation with antibody-fusion proteins and bispecific antibody-mediated T cell response
Source: Cancer Immunol Immunother. 2020 Jun 5;69(11):2291–303. doi: 10.1007/s00262-020-02624-6 (PMC7568714; doi:10.1007/s00262-020-02624-6)
Supplement: Supplementary file 2 — Supplementary file2 (PDF 72 kb) [file 262_2020_2624_MOESM2_ESM.pdf]

Supplementary Table 2

Fusion protein sequences

Fusion protein	Sequence
**scFvEGFR-4-1BBL**	EVQLVESGGGLVQPGGSLRLSCAASGFSLTNYGVHWVRQAPGKGLEWLGVIWSGGNTDYN<br>TPFTSRFTISRDNSKNTLYLQMNSLRAEDTAVYYCARALTYYDYEFAYWGQGTTVTVSSGGG<br>GSGGGGSGGGGSDIQLTQSPSFLSASVGDRVTITCRASQSIGTNIHWYQQKPGKAPKLLIKYA<br>SESISGVPSRFSGSGSGTEFTLTISSLQPEDFATYYCQQNNNWPTTFGAGTKLEIKRAAAHHH<br>HHHGGGGSREGPELSPDDPAGLLDLRQGMFAQLVAQNVLLIDGPLSWYSDPGLAGVSLTG<br>GLSYKEDTKELVVAKAGVYYVFFQLELRRVVAGEGSGSVSLALHLQPLRSAAGAAALALTVDL<br>PPASSEARNSAFGFQGRLLHLSAGQRLGVHLHTEARARHAWQLTQGATVLGLFRVTPEIPAG<br>LPSPRSE
**scFvEGFR-OX40L**	EVQLVESGGGLVQPGGSLRLSCAASGFSLTNYGVHWVRQAPGKGLEWLGVIWSGGNTDYN<br>TPFTSRFTISRDNSKNTLYLQMNSLRAEDTAVYYCARALTYYDYEFAYWGQGTTVTVSSGGG<br>GSGGGGSGGGGSDIQLTQSPSFLSASVGDRVTITCRASQSIGTNIHWYQQKPGKAPKLLIKYA<br>SESISGVPSRFSGSGSGTEFTLTISSLQPEDFATYYCQQNNNWPTTFGAGTKLEIKRAAAHHH<br>HHHGGGGSQVSHRYPRIQSIKVQFTEYKKEKGFILTSQKEDEIMKVQNNSVIINCDGFYLISLK<br>GYFSQEVNISLHYQKDEEPLFQLKKVRSVNSLMVASLTYKDKVYLNVTTDNTSLDDFHVNGG<br>ELILIHQNPGEFCVL
**B7-DbEGFR**	VIHVTKEVKEVATLSCGHNVSVEELAQTRIYWQKEKKMVLTMMSGDMNIWPEYKNRTIFDI<br>TNNLSIVILALRPSDEGTYECVVLKYEKDAFKREHLAEVTLSVKADFPTPSISDFEIPTSNIRRIICS<br>TSGGFPEPHLSWLENGEELNAINTTVSQDPETELYAVSSKLDFNMTTNHSFMCLIKYGHLRV<br>NQTFNWNTTKQEHFPDNGGGSGGGSGGGSGGGSSGGGSEVQLVESGGGLVQPGGSLRLS<br>CAASGFSLTNYGVHWVRQAPGKGLEWLGVIWSGGNTDYNTPFTSRFTISRDNSKNTLYLQM<br>NSLRAEDTAVYYCARALTYYDYEFAYWGQGTTVTVSSGGGGSDIQLTQSPSFLSASVGDRVTI<br>TCRASQSIGTNIHWYQQKPGKAPKLLIKYASESISGVPSRFSGSGSGTEFTLTISSLQPEDFATYY<br>CQQNNNWPTTFGAGTKLEIKRAAAHHHHHH
