# Supplementary material for: Influence of antigen density and immunosuppressive factors on tumor-targeted costimulation with antibody-fusion proteins and bispecific antibody-mediated T cell response
Source: Cancer Immunol Immunother. 2020 Jun 5;69(11):2291–303. doi: 10.1007/s00262-020-02624-6 (PMC7568714; doi:10.1007/s00262-020-02624-6)
Supplement: Supplementary file 3 — Supplementary file3 (PDF 111 kb) [file 262_2020_2624_MOESM3_ESM.pdf]

Supplementary Fig.1

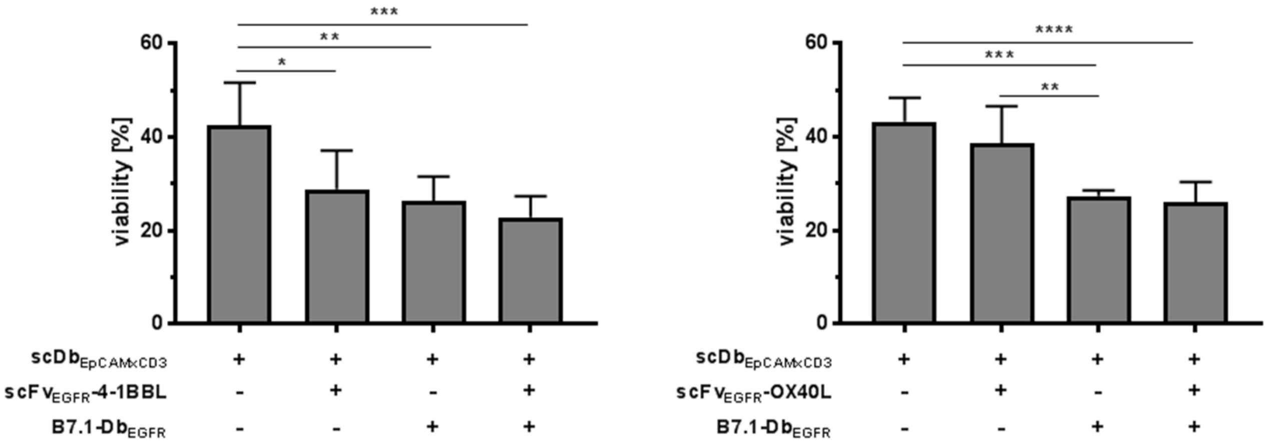

Suppl.Fig.1 scDbEpCAMxCD3-mediated tumor cell killing was enhanced by costimulated PBMC. PBMC were prestimulated with 1 pM scDbEpCAMxCD3 +/- 10 nM of one or two of the indicated costimulatory fusion proteins for 6 days in co-culture with Lovo cells. Subsequently, the preactivted PBMC were added to freshly seeded Lovo cells at the ratio 5:1 and incubated with (A) 0.4 nM and (B) 2 nM scDbEpCAMxCD3 for 6 h. Afterwards, PBMC were removed and tumor cell viability measured by MTT assay. Graphics show mean ± SD, n=3, (duplicates in each assay), blockshift correction, One-way ANOVA, Tukey's post-test, *, p<0.05; **, p<0.01; ***, p<0.001, **** p<0.0001
